# Web-based therapeutic exercise resource center as a treatment for knee osteoarthritis: a prospective cohort pilot study

**DOI:** 10.1186/1471-2474-15-158

**Published:** 2014-05-17

**Authors:** M Alison Brooks, John E Beaulieu, Herbert H Severson, Christa M Wille, David Cooper, Jeff M Gau, Bryan C Heiderscheit

**Affiliations:** 1 University of Wisconsin-Madison, 1685 Highland Ave, Madison, WI 53705, USA; 2 Visual Health Information, Inc., 11003 A St. South, Tacoma, WA 98444, USA; 3 Oregon Research Institute, 1776 Millrace Drive, Eugene, OR 97403, USA; 4 University of Wisconsin-Madison, 4195 Medical Sciences Center, 1300 University Ave, Madison, WI 53706, USA; 5 University of Wisconsin-Madison, 4120 Medical Sciences Center, 1300 University Ave, Madison, WI 53706, USA

**Keywords:** Knee osteoarthritis, Internet, Home exercise program

## Abstract

**Background:**

Although beneficial effects of exercise in the management of knee osteoarthritis (OA) have been established, only 14 -18% of patients with knee OA receive an exercise from their primary care provider. Patients with knee OA cite lack of physician exercise advice as a major reason why they do not exercise to improve their condition. The purpose of this pilot study was to investigate use of a web-based Therapeutic Exercise Resource Center (TERC) as a tool to prescribe strength, flexibility and aerobic exercise as part of knee OA treatment. It was hypothesized that significant change in clinical outcome scores would result from patients’ use of the TERC.

**Methods:**

Sixty five individuals diagnosed with mild/moderate knee OA based on symptoms and radiographs were enrolled through outpatient physician clinics. Using exercise animations to facilitate proper technique, the TERC assigned and progressed patients through multiple levels of exercise intensity based on exercise history, co-morbidities and a validated measure of pain and function. Subjects completed a modified short form WOMAC (mSF-WOMAC), World Health Organization Quality of Life (WHO-QOL) and Knee Self-Efficacy Scale (K-SES) at baseline and completion of the 8 week program, and a user satisfaction survey. Outcomes were compared over time using paired t-tests and effect sizes calculated using partial point biserial (*pr*).

**Results:**

Fifty two participants completed the 8 week program with average duration of knee pain 8.0 ± 11.0 yrs (25 females; 61.0 ± 9.4 yrs; body mass index, 28.8 ± 6.3 kg/m^2^). During the study period, all outcome measures improved: mSF-WOMAC scores decreased (better pain and function) (*p* < .001; large effect, *pr* = 0.70); WHO-QOL physical scores increased (*p* = .015; medium effect, *pr* = 0.33); and K-SES scores increased (*p* < .001; large effect, *pr* = 0.54). No significant differences were found in study outcomes as a function of gender, age, BMI or symptom duration. Patients reported very positive evaluation of the TERC (94% indicated the website was easy to use; 90% specified the exercise animations were especially helpful).

**Conclusion:**

This pilot study demonstrated the web-based TERC to be feasible and efficacious in improving clinical outcomes for patients with mild/moderate knee OA and supports future studies to compare TERC to current standard of care, such as educational brochures.

## Background

Knee osteoarthritis (OA) is the most common joint disorder, with the lifetime risk of developing symptomatic knee OA estimated to be as high as ~45% (40% men and 47% women) [1]. As one of the five leading causes of disability in adults over the age of 50 yrs [2], knee OA is a significant threat to their quality of life and independence [3]. Given the forecasted growth in the U.S. older adult population, the prevalence of knee OA is expected to rise dramatically over the next several decades [4], highlighting the need for effective disease management strategies.

Over the past 15 years a substantial body of literature has emerged supporting the beneficial effects of using exercises in the management of knee OA [5-7]. Multiple randomized controlled trials have observed reduced pain and increased function with the use of muscle strengthening and joint range of motion exercises in individuals with knee OA even with only moderate adherence [8-10]. The use of exercises is recommended in a number of recent national and international clinical practice guidelines for the non-arthroplasty management of those with OA of the knee [11-16].

Management of knee OA with exercise is considered to be the corner-stone of conservative self-management for this chronic disease [7]. Despite the substantial body of literature supporting this recommendation, the large majority of physicians do not incorporate exercise as part of their management plan for these patients; overall, the use of exercise by physicians to manage musculoskeletal problems is very low: 14% [17]. The major factors that account for this non-use of exercise prescription are: limited contract time with the patient, lack of training in medical school resulting in an inadequate knowledge base about exercises, and lack of appropriate support materials [18-21]. Patients with OA cite the lack of exercise advice from their physician, as it relates to arthritis, as a major reason they do not exercise to improve their condition [21].

The Internet can be successfully used as medium for providing self-management and rehabilitation interventions for knee OA [22], with individuals receiving individualized and interactive guidelines reporting improved pain, stiffness and physical function, as well as being satisfied with this approach [23-25]. The use of the Internet for delivery of health and activity interventions also has the potential to lower the cost of delivery and improve user acceptance and satisfaction. There are many barriers associated with in-person training that patients face, such as distance, cost of travel, time away from work or caregiver duties, cost of care if under- or un-insured, and limited access to healthcare providers in rural areas. These barriers can be overcome by Internet-based delivery systems [26].

Although internet-based physical activity resources are currently available, many are not geared toward patients with lower extremity OA or do not include patient-specific tailoring based on consideration of pain and functional limitations. As such, we developed the Therapeutic Exercise Resource Center (TERC), the first comprehensive web based system designed to evaluate, prescribe, monitor and adjust therapeutic exercise programs for patients with knee OA. Based on patient-entered information, an individualized routine containing strength, flexibility and aerobic exercises is generated and progressed to target condition-specific neuromuscular impairments and improve knee OA symptoms and overall health. The TERC can be used by health care providers to promote exercise in patients with knee OA. The purpose of this pilot study was to determine if the use of the TERC as part of management for knee OA would improve patient-reported clinical outcome measures.

## Methods

### Study design

Prospective, cohort study with 8 week follow-up for each participant. This study was approved by the University of Wisconsin Health Sciences Institutional Review Board.

### Participants

Individuals diagnosed with mild to moderate knee OA were recruited, with diagnosis made by their physician based on symptoms (experiencing knee pain on most days) and plain radiographs as outlined by the American College of Rheumatology Clinical plus Radiographic Classification Criteria for knee OA [27]. The radiographs had been obtained as part of the patient’s routine care, not specific to this study. Once patients were identified as potentially eligible, their medical record was reviewed, including plain radiographs, to insure that they met radiographic classification criteria for mild/moderate knee OA. Additional inclusion criteria included: age 25 years or older; living independently; considered themselves in general good health; able to walk without an assistive device; able to speak and read English; have a home computer with an Internet connection; have a personal e-mail account; and able to attend at least four online visits to the TERC web site. Exclusion criteria included currently under the care of a physical therapist, a fall more than 2 times in past 6 months, knee injection within past 4 weeks or scheduled in next 8 weeks, and diagnosis or symptoms of: terminal illness; unstable angina, congestive heart failure, uncontrolled hypertension, orthostatic hypotension; emphysema, chronic obstructive pulmonary disease; Parkinson’s disease, stroke, brain disease, peripheral neuropathy; crystalline or inflammatory arthritis; and knee joint replacement.

Patients were recruited through primary care or specialty physician clinics of University of Wisconsin Hospital and Clinics in Madison, WI, USA. Potentially eligible patients were identified from standard medical visits or informational opt-in letters. Patients identified during clinic visits provided their contact information to the participating physician, and were then contacted by the research staff. Interested patients receiving the opt-in letters contacted the research staff directly to complete a phone screen and determine eligibility. All participating patients provided informed written consent.

### Procedures

Following enrollment, patients were directed to use their personal computer to visit the TERC web site’s main page (accessible for study only; currently not publicly available) where they entered a unique code given to them by a study investigator that allowed access to the website. Each patient then created their own account with a user name and password, and began interacting with the TERC features. The TERC provided individualized exercise routines for each patient, as well as general educational information about knee OA. Some exercises required the use of elastic resistance tubing or ankle weights (one pair of 2.27 kg weights adjustable in increments of 0.45 kg), which were mailed to the patients with instructions for use at the start of the 8-week program. Patients were instructed to exercise daily and record their exercise frequency using the TERC exercise log. Outcomes were assessed through online questionnaires at baseline and 8-weeks.

### TERC intervention

The exercises within the TERC were comprised of strength and flexibility components with recommendations for aerobic activities, consistent with current standard of care and clinical practice guidelines [12,16,28-30]. Strength and flexibility exercises primarily targeted the quadriceps, hamstrings and gluteal muscles, while the aerobic recommendations were comprised of a progressive walking program. All exercises were to be performed five times per week. To allow individualization of exercise prescription, patient-entered responses to online questionnaires including the modified short form Western Ontario and McMaster Universities Arthritis Index ( mSF-WOMAC) [31], exercise history and general health information were used to determine the initial level of exercise difficulty. Individuals having greater symptoms, functional limitations and less exercise experience were assigned a less difficult routine. Exercise routine difficulty was modified by the number of assigned strengthening and flexibility exercises (total of 4 or 5); the resistance of the strengthening exercises (i.e., body weight, ankle weights or elastic resistance tubing); and the duration and speed of walking.

**Figure 1:**
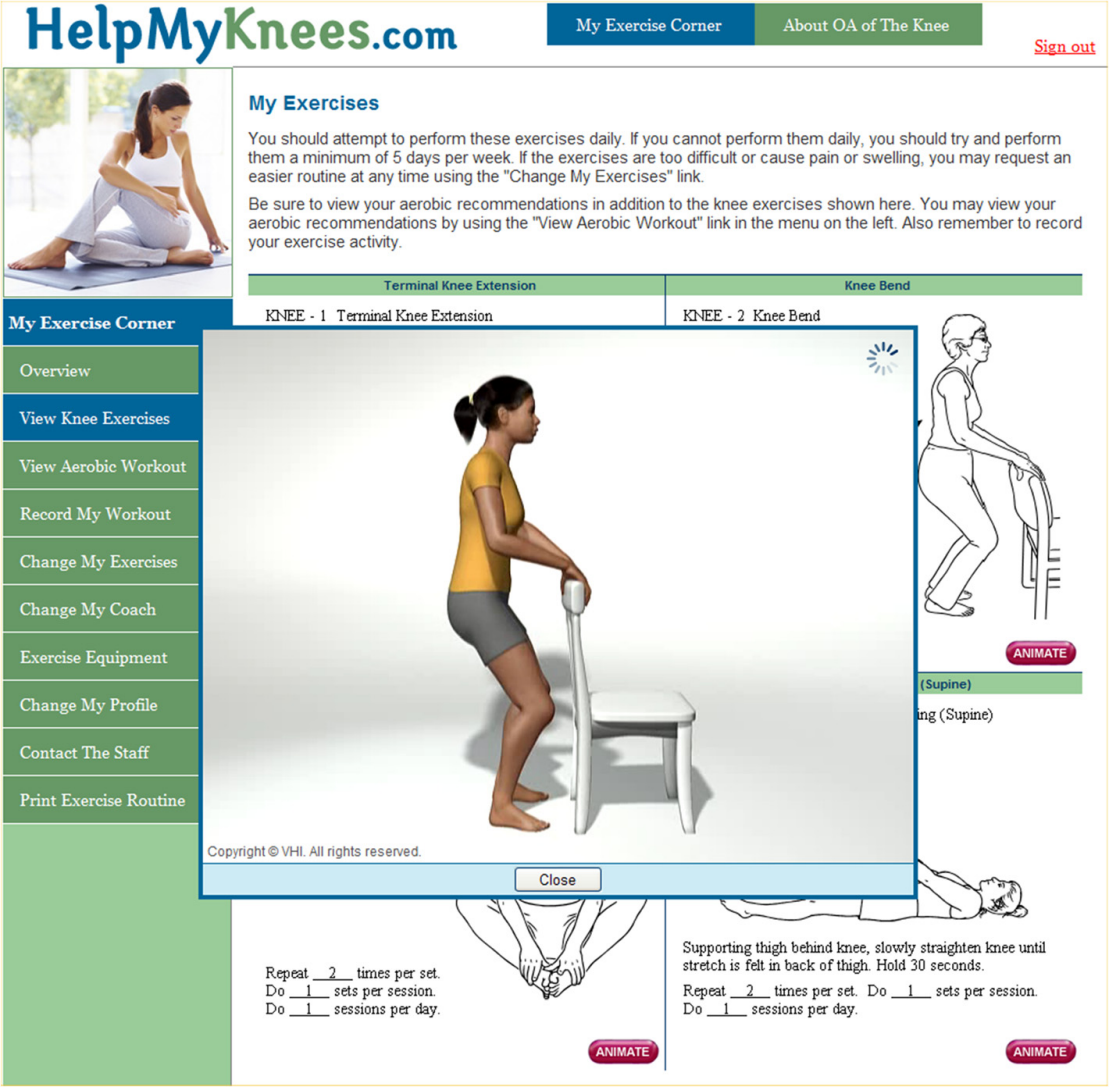
Screen capture of the TERC website displaying line drawing exercises and an accompanying animation.

The TERC displayed static images of each individualized exercise routine, as well as motion captured animations of each strength and flexibility exercise to promote correct technique (Figure 1). Participants were given the option to print a copy of their daily workout to serve as a reference for use away from the computer. The TERC also contained evidence-based educational information to help patients better understand knee OA risk factors, pathogenesis and symptom management. After completing the daily exercise program, patients were asked to record via online logs if they completed the prescribed exercises and if they experienced an increase in knee pain. If participants did not record in the online logs for a one week period, they received an automated message from the TERC reminding them of the importance of exercising and of recording their exercise information.

At any time during the 8-week period, patients were able to change their exercise routine to one that was more or less difficult. When a more difficult routine was requested, the patient was prompted to complete the mSF-WOMAC. If the patient demonstrated a score equal to or better than the prior score, a more difficult routine was created for the patient; otherwise, new exercises having the same degree of difficulty as the previous routine were selected. When a patient opted for a less difficult routine, one was immediately created for them.

Throughout the study, the TERC monitored each participant’s activity and exercise logs and would send an automated notification to the participant if certain criteria were met. Patients that reported exercising for 2 weeks without an increase in pain were automatically prompted by the TERC to consider a more difficult exercise routine. Patients that recorded 3 or more consecutive days of increased knee pain were automatically prompted by the TERC to consider a less difficult routine. If increased knee pain continued, study personnel were notified. Patients could also communicate with study personnel via the TERC, and request a reply if needed. Any questions pertaining to exercises or symptoms were handled by a healthcare professional associated with the study (i.e., physician or physical therapist).

#### Primary outcome measures

Knee pain, stiffness and daily function were assessed using the mSF-WOMAC, found to be a valid, reliable and responsive alternative to the traditional WOMAC in the evaluation of patients with knee OA managed conservatively [31]. Response options were on a 5-point scale (0 = none, 4 = extreme). A lower score represents better function. Good internal consistency was observed in the current study (Cronbach’s alpha = .92). In addition, a global rating of knee symptom change was determined with a single 5-point Likert scale (0 = a lot worse, 5 = a lot better).

Quality of life was assessed with a shortened version of the World Health Organization Quality of Life scale (WHO-QOL) [32]. The short version measured quality of life in the areas of psychological health (6-items; e.g., How much do you enjoy life?) and physical health (7-items; e.g., Do you have enough energy for everyday life?). Response options were on a 5-point scale (e.g., 1 = not at all, 5 = an extreme amount). A higher score represents better quality of life. Good internal consistency was found in the current study for the physical health and psychological health scales (Cronbach’s alphas = .87 and .84, respectively).

#### Secondary outcome measures

Self-efficacy to perform activities was assessed with 7-items from the daily activities subscale of the Knee Self-Efficacy Scale (K-SES; [33]. Participants were asked on a 5-point scale (1 = not at all comfortable, 5 = very comfortable) how certain they were about performing daily activities (e.g., climbing up and down stairs). A higher score represents higher self-efficacy. Good internal consistency was found in the current study (Cronbach’s alpha = .88). Following the 8-week intervention, participants completed a Global Rating of Change scale (GRC) which asked them to rate their change in knee OA symptoms using a 5-point scale (“a lot worse,” “somewhat worse,” “no change,” “somewhat better,” “a lot better”). User satisfaction was assessed at the 8-week follow-up with an 18-item questionnaire that asked about: a) the TERC’s perceived ease of use and perceived usefulness; b) level of satisfaction with TERC; c) opinions regarding the exercises prescribed by TERC and d) the likelihood of using a web-based TERC program and performing the exercise routines. Each question was answered on a 5-point scale (0 = strongly disagree, 5 = strongly agree). An overall score of user satisfaction was computed as the mean across all 18 items with the last two items reverse scored due to question formatting.

### Statistical analysis

Basic demographic variables were collected at baseline, including age, gender, weight, height, BMI, and years of education. Descriptive statistics were calculated for demographic variables and metrics of website access and use by the participants. Prior to analysis all measures were screened for normality and no significant violations found. The main analysis examined pre- to post-training changes with respect to physical functioning, health-related quality of life, and self-efficacy toward performing the exercise routines. Analyses were conducted on participants with complete datasets. A within-subject, repeated measures approach was used to test for change in the measures by comparing the baseline scores obtained at the pre-training assessments to scores obtained during the post-training assessments. Paired t-tests were used to test the hypothesis that average change in study outcomes from baseline to the 8-week follow-up assessment was statistically greater than zero. A partial point biserial *r* (*pr*) was computed as the measure of effect size and is based on t-value and degrees of freedom [34]. Outcome differences were also examined with respect to patient characteristics (gender, age, education level, BMI, duration of knee pain) by including a between-subjects factor into the repeated measures ANOVA models examining baseline to 8-week follow-up scores.

The GRC was used to assess the minimum clinically important difference (MCID) for change in the short form knee function scale. The GRC was dichotomized into “no improvement” (corresponding to answers of “a lot worse,” “somewhat worse” or “no change”) and “improvement” (“somewhat better” or “a lot better”). Independent t-tests were used to compare change in the short form knee function scale from baseline to the 8-week follow-up between participants reporting no improvement and those reporting improvement on the GRC.

To bolster our confidence in the internal validity of this quasi-experimental design, we tested the hypothesis that participants who reported greater satisfaction with the program would show greater improvement in pre-post scores. Regression models were used to examine residual scores at the 8-week follow-up assessment with the baseline score as a covariate and the overall satisfaction score as a predictor.

With a two-tailed alpha set to 0.05, 37 participants were needed to achieve 0.80 power to detect medium size effects or larger (*d*_*z*_ = 0.48) for pre-post change, as well as to have 0.80 power to detect *r* =0 .44 or larger correlation coefficients. To account for possible drop outs and missing data, an additional 28 participants were enrolled.

## Results

**Figure 2:**
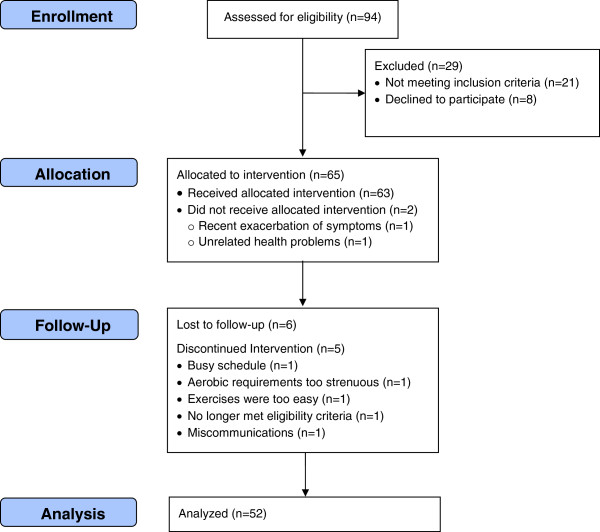
Flow diagram of participant enrollment.

Of the 65 patients enrolled, 52 completed both the baseline and 8-week follow-up assessment and were used in the analyses (Figure 2). The thirteen patients that did not complete both assessments were mostly male (53%), had a mean age of 62.6 ± 9.9 years, and a mean BMI of 35.4 ± 11.53 kg/m^2^. Sex and age did not statistically differ between the two groups, however, those that did not complete both assessments had significantly higher BMI scores then those that did (t [58] = 2.61, p = 0.011). For the 52 participants that completed the study, the average duration of knee pain was 8.0 ± 11.0 yrs. At the start of the study, 41 subjects reported having prior physical therapy for knee OA, and 21 subjects reported continuing exercises prescribed by their physical therapist to some degree. Participants (age, 61 ± 9.4 years; weight, 85.8 ± 20.1 kg; BMI, 28.8 ± 6.3 kg/m^2^) were approximately half female with 63% having a four-year college degree or greater (Table 1). All patients logged in to the TERC and did so on 47.2 ± 12.0 days (range, 9–56) out of the possible 56 program days. Exercise animations were viewed by 48 patients, with 17.3 ± 21.9 animations being viewed (Table 2). Patients on average reported performing knee exercises on 37.8 ± 12.5 days (range, 9–56) and aerobic exercise on 35.1 ± 12.8 days (range, 5–56). Sixty-five percent of the patients requested a more difficult exercise routine at some point during the 8-week intervention, while only 15.4% requested an easier routine (Table 2). No exercise-related injuries were reported.

**Table 1:** Participant characteristics

	**N**	**%**
Female	25	48.1
Age (yrs)		
33 – 45	3	5.8
46 – 50	4	7.7
51 – 55	11	21.2
56 – 60	10	19.2
61 – 65	10	19.2
66 – 70	9	17.3
71 – 75	3	5.8
76+	2	3.8
BMI (kg/m^2^)*		
Underweight (<18.5)	0	0.0
Normal (18.5-24.9)	14	28.0
Overweight (25.0-29.9)	20	40.0
Obese (≥30)	16	32.0
Highest education level		
High school/GED	3	5.8
Some college	7	13.5
2-Year college degree	9	17.3
4-Year college degree	17	32.7
Master degree	10	19.2
Doctoral degree	6	11.5

*BMI categories: <18.5 Underweight; 18.5-24.9 Normal; 25.0-29.9 Overweight; ≥30 Obese.

**Table 2:** Participant interaction with TERC

		**Times the activity was performed per participant**	
	**n**	**Mean**	**Range**
*Patient initiated communication*			
Submitted comment or question to study staff	33	1.7	(0, 4)
Requested a reply from staff to submitted question	20	1.3	(0, 2)
*TERC initiated communication*			
Exercises may be too easy	34	1.4	(0, 4)
Exercises may be too hard	15	2.1	(0, 6)
Pain warning sent to study personnel	6	1.0	(0, 1)
Stopped recording exercise	41	2.6	(0, 7)
*Patient usage of TERC features*			
Viewed exercise routine	52	16.7	(2, 56)
Viewed exercise animations	48	18.8	(0, 123)
Viewed aerobic recommendations	52	12.3	(1, 52)
Printed exercise routine	18	1.6	(0, 3)
Requested less difficult exercise routine	8	1.3	(0, 2)
Requested more difficult exercise routine	34	2.7	(0, 7)
Denied request for more difficult exercise routine based on mSF-WOMAC	4	1.8	(0, 3)

n, number of patients that performed the activity (total of 52).

All of the patient-reported clinical outcome measures improved over the 8 week study period (Table 3). Knee function (mSF-WOMAC) scores significantly decreased (overall better function) with a large effect size (*pr* = 0.70). All three subscale scores also significantly decreased (indicating decreased pain and stiffness and improved daily functioning) with large effect sizes. Knee self-efficacy scores significantly increased with a large effect size *(pr* = 0.54). WHO-QOL physical scores significantly increased with a medium effect size (*pr* = 0.33), and although the increase in WHO-QOL psychological scores was not statistically significant, the change was in the hypothesized direction and associated with a small effect size (*pr* = .18). Analysis of outcomes by patient characteristics were all non-significant suggesting the program worked equally well for all across gender, age, education level, BMI, and duration of symptoms.

**Table 3:** Changes in self-reported clinical outcomes toolsSD = standard deviation; pr = point-biserial partial regression coefficient as measure of effect size with 0.14 small, 0.36 medium, and 0.51 large effect (Rosnow & Rosenthal, 2008)

	**Baseline**		**8-Weeks**		**Test statistics**		
	**Mean**	**SD**	**Mean**	**Mean**	**SD**	**Mean**	**Mean**
mSF-WOMAC	17.00	7.67	10.42	7.94	7.01	<0.001	0.70
Stiffness	3.23	1.55	1.96	1.40	6.66	<0.001	0.68
Pain	5.67	2.60	3.60	2.91	6.20	<0.001	0.66
Daily functioning	8.10	3.99	4.87	4.10	6.51	<0.001	0.67
WHO-physical	67.28	15.87	72.21	14.44	-2.53	0.015	0.33
WHO-psychological	74.06	12.93	75.90	13.76	-1.28	0.208	0.18
Self-efficacy	16.94	4.77	19.59	6.06	-4.60	<0.001	0.54

The distribution of responses on the GRC showed 4% (n = 2) of patients reported their measure of change as “somewhat worse”, 16% (n = 8) “no change”, 51% (n = 26) “somewhat better” and 29% (n = 15) “a lot better”. One patient did not complete the GRC. The mean change in the short form knee function scale from baseline to 8-week follow-up was -7.61 ± 6.93 for those reporting “improvement” (n = 41, 80%) and -2.70 ± 4.81 for those reporting “no improvement” (n = 10, 20%). Mean differences compared using independent t-tests were statistically significant (t [49] = 2.11, p = .040).

The overall satisfaction score was 3.1 ± 0.5 indicating participants found a high degree of satisfaction with the program;with the majority reporting that the website helped them better understand their knee OA condition (Table 4). Patients reported very positive evaluation of the TERC, with 94% indicating the website was easy to use and 90% specifying the exercise animations were especially helpful. The TERC procedure for placing patients at the appropriate level of the exercise continuum was effective, with 78.8% reporting that the assigned exercise routine was appropriate and only 1.9% of patients reporting that the initially assigned exercises were too hard. Almost half of the patients reported taking less pain medication since they began doing the exercises. Over two-thirds of patients felt that the website content helped them better understand and manage their condition.

**Table 4:** Program satisfaction ratings

	**Percent selecting each category (%)**					**Mean**	**SD**
	**0**	**1**	**2**	**3**	**4**		
The overall organization of the site is easy to understand.	0.0	1.9	3.8	50.0	44.2	3.4	0.7
Individual pages are well designed.	0.0	1.9	9.6	48.1	40.4	3.3	0.7
It was easy to move from one page to another.	0.0	3.8	3.8	48.1	44.2	3.3	0.7
Terminology used in the website was clear	0.0	1.9	3.8	50.0	44.2	3.4	0.7
The content of the website helped me better understand OA of the knee.	3.8	5.8	13.5	44.2	32.7	3.0	1.0
My assigned exercise routines were appropriate for me.	1.9	1.9	17.3	53.8	25.0	3.0	0.8
The ability to request a harder or easier routine was helpful.	0.0	3.8	11.5	53.8	30.8	3.1	0.8
The information on the web site helped me to better manage my condition.	0.0	5.8	26.9	34.6	32.7	2.9	0.9
I was able to complete my tasks in a reasonable amount of time.	0.0	1.9	9.6	63.5	25.0	3.1	0.7
The e-mails reminding me to record my exercise workouts were helpful.	0.0	1.9	9.6	51.9	36.5	3.2	0.7
I am taking less pain medication since I began doing exercises.	5.8	15.4	34.6	28.8	15.4	2.3	1.1
The animated exercise illustrations were helpful.	0.0	3.8	5.8	40.4	50.0	3.4	0.8
The animated exercises were easy to follow.	1.9	0.0	5.8	44.2	48.1	3.4	0.8
The daily exercise recording tool was easy to use.	0.0	1.9	5.8	38.5	53.8	3.4	0.7
Overall, the website is easy to use.	0.0	0.0	5.8	40.4	53.8	3.5	0.6
I would likely use the website in the future.	1.9	5.8	11.5	34.6	46.2	3.2	1.0
The initially assigned exercises were too easy.*	1.9	15.4	26.9	34.6	19.2	2.6	1.1
The initially assigned exercises were too hard.*	40.4	28.8	26.9	0.0	1.9	0.9	0.8

0 = strongly disagree, 1 = disagree, 2 = neutral, 3 = agree, 4 = strongly agree.

SD = standard deviation.

*disagreement (category 0 and 1) is a favorable response.

Results of the residual gain score analysis showed that greater reports of program satisfaction resulted in trend level or significant effects for all study outcomes. Greater reports of program satisfaction were associated with lower short form knee function scale scores (*t* = -1.99, *p* = .052, *r* = -.26), higher WHO psychosocial health scores (*t* = 3.27, *p* = .002, *r* = .28), higher WHO physical scores (*t* = 3.50, *p* = .001, *r* = .36), and higher self-efficacy scores (*t* = 2.46, *p* = .017, *r* = .32). The average effect across all outcomes (*r* = .31) was associated with a medium effect size.

## Discussion

This pilot study demonstrated the web-based TERC to be both feasible and efficacious in improving clinical outcomes for patients with mild to moderate knee OA. After an online eight week training program, patients reported improved physical function, pain, and stiffness; improved quality of life related to physical health; and improved self-efficacy to perform daily activities. Four out of five patients stated they experienced an improvement in their knee symptoms after beginning the program, with nearly half reporting decreased use of pain medication. Overall user satisfaction with the web-based TERC was high with 80% of patients agreeing to use the website in the future.

Any intervention that improves physical function in patients with knee OA can have a significant public health impact. For community-dwelling adults aged 50 years and older, knee OA is the leading cause of disability during walking, stair climbing and daily activities [35]. This resulting decline in functional mobility places an undue burden on the aging population and seriously threatens their ability to live independently [3]. Our results are consistent with previous studies demonstrating that exercising can lessen pain, reduce stiffness, and improve physical functioning even with only moderate adherence [9,10,36]. Therefore it should be standard of care for patients with knee OA to be prescribed an exercise program for ongoing self-management of their chronic condition.

Supervised exercise programs for knee OA are often felt to achieve better outcomes in terms of reduction of pain and short-term incapacity compared to non-supervised programs [37].

However, this effect appears to be more related to the number of supervised visits, with a greater improvement in outcomes observed with 12 or more supervised visits [35]. In contrast, several investigations have found videotape exercise instruction to have similar benefits to in-person instruction [38-40]. A computer-aided exercise training program was shown to be comparable to direct care at improving functional status, activities of daily living, pain, and range of motion in patients following total knee arthoplasty [41]. Further, proper exercise technique has been shown to be achievable through computer-aided instruction [40,42]. This study’s findings support that patients with knee OA can experience significant improvements in self-reported physical function during daily activities using a computer-aided exercise program. This is important when considering patient-specific barriers that limit an individual’s ability to receive supervised physical therapy. For example, underserved populations living in more rural areas may not have access to a physical therapist without traveling a considerable distance. In addition those working full-time or providing care for an elderly dependent may be unable to seek care for themselves. A computer-aided exercise program would help overcome these barriers and provide these patients with an effective treatment that they might not otherwise receive. Additionally the range of participants in our study illustrates that an internet-delivered intervention is possible across age and educational levels.

Exercise adherence is critical for the short and long-term success of exercise therapy to treat knee OA [5]. Adherence to a recommended exercise regimen may be higher when a patient is asked to record the results of their daily exercise session [43,44] or is asked about their compliance at follow-up visits with a therapist or physician. [45-47]. In this study patients were asked to record the results of their daily exercise session on the TERC, and reported compliance with the recommended exercise regimen showed very high engagement with the program, with patients reporting they performed knee exercises on average 4–5 times per week. In addition this web-based intervention was unique in that it was tailored to each individual patient based on their self-reported pain level and function. It is possible that the high adherence rates may be due to the fact that patients were given an appropriate level of exercise difficulty that controlled for their baseline level of pain and function.

It was very encouraging that our moderation analysis showed patients who were older, had greater BMI, or had a longer duration of symptoms reported similar improvements in their pain, physical function, quality of life, and self-efficacy as those who were younger, had lower BMI, and had shorter duration of symptoms. This may in part illustrate that the TERC placed patients at an exercise level that they could successfully perform while allowing for exercise progression as patients improved. The majority of patients reported that the exercises they were assigned were not too hard and that it was helpful to be able to request a harder routine when they felt ready. Conversely it is possible that younger patients with less pain and better function at baseline would have less room for improvement on the self-reported outcome measures. However this study showed that the TERC-prescribed program was equally effective in younger patients with less baseline morbidity. These findings are not surprising as exercise has been shown to be an effective front line treatment for OA of the knee in addition to being effective for those with worsening of the condition [5,6,48].

Unfortunately a low percentage (12-19%) of physicians either prescribe exercise or refer patients to a physical therapist as part of their management for knee OA [49,50]. Mirand et al. created a set of guidelines for the development of a practice intervention tool that could be used to change patient health behaviors [51]. They concluded that in order for physicians to use an intervention tool it must: 1) be able to be used within the limited office time of 3–5 minutes; 2) take into account the lack of physician skills; 3) produce assessments and recommendations that are tailored to the patient's condition; and 4) be a web-based tool that is maintained and updated by an outside organization which would be responsible for providing information that is scientifically accurate. Although Mirand and colleagues were not directly addressing the issue of exercise prescription, many of their recommendations address the major barriers that prevent physicians from prescribing exercise as a management option for musculoskeletal conditions including knee OA [51]. The need to reduce referrals to specialists and reduce health care costs highlights the importance of the primary care physician being able to directly assist patients with their management for OA conditions. The TERC eliminates the need for physician knowledge about specific exercise routines by placing the patient in an appropriate exercise program based on patient-specific variables. The TERC simply requires the physician to provide the patient with web access and can be readily used as an important initial or adjunct treatment for patients with mild to moderate knee OA.

When one considers the limited patient contact time, reduced comfort with exercise prescription, and lack of adequate support materials that physicians have identified as barriers within their practice, it is possible that patients with OA of the knee will not receive adequate instruction from their physician regarding proper exercise performance. TERC provides patients with high-quality animations of people demonstrating correct modeling of therapeutic exercises for knee OA. Live modeling or video instruction of exercises has been found to substantially reduce the number of performance errors compared to the use of printed exercise handouts alone [38,52]. Our prior work found that the use of animations to instruct patients in exercise performance increased the probability that those exercises would be executed correctly [53]. Patients’ confidence in their ability to correctly perform prescribed exercises increases the probability of compliance to the exercise regimes [54,55]. In this study patients on average viewed the animations over 50 times during the 8 week study period, and over 90% of patients reported that the animated exercises were helpful and easy to follow.

Strengths of this pilot study were its ability to show significant improvements in knee physical function and self-efficacy and good patient compliance with the assigned exercise routine. In addition the web-based TERC is designed to be an ongoing intervention tool which patients can use for lifelong management of their knee OA, as opposed to supervised care which usually involves a very limited number of visits over a relatively short duration. Limitations of the study were not having a control condition and small sample size. The lack of a control group limits the conclusions that can be drawn from the results as we are unable to compare the outcomes from the TERC to other exercise-based treatment options currently available for the management of mild to moderate knee OA. For the purposes of the research study, the eligibility criteria eliminated lower functioning patients such as those with a history of falls or walking with an assistive device, and thus the findings may not be generalizable to all patients with mild/moderate knee OA. Similarly, because MCID values will vary based on the population studied and the chosen calculation methodology, the MCID we report for the mSF-WOMAC may not be generalizable. In addition some patients had prior experience with supervised physical therapy and reported doing doing some of their prescribed exercises at the start of the current study. Nonetheless, many of these individuals reported improved symptoms once beginning their TERC-prescribed exercise routine.

Thirteen of the 65 patients (20%) did not complete the 8-week follow-up questionnaire, with six being lost to follow-up. It is possible that these 13 patients did not improve or were not satisfied with the internet-based intervention. As program usage is associated with drop-out, it is also possible that adherence rates in this study may be overestimated [56]. We cannot directly ensure that participants performed the exercises correctly. However there is substantial published evidence that direct clinician-patient interaction is not necessary to achieve effective exercise instruction [38-42]. While the majority of patients in this cohort reported symptomatic improvement on the global rating of change scale, it is possible that the 10 patients who reported no change or slightly worse symptoms did the exercises wrong. Finally, because of the duration of our study (8 weeks), we are unable to determine if the positive clinical outcomes would persist over a longer follow-up period when patient adherence is likely to decrease.

## Conclusion

In this pilot study, patients with mild to moderate knee OA who used the TERC for 8 weeks experienced significant improvement in self-reported clinical outcome measures, including pain, physical function, quality of life, and self-efficacy. Positive clinical attributes of the TERC program include the feasibility and efficacy of the individualized exercise regimen developed for each participant, as well as the overall satisfaction participants reported. Given the significant positive clinical outcomes of this non-randomized one arm pilot study, future studies may include a randomized controlled trial, and we expect to move forward with a larger trial comparing web-based TERC to more traditional office-based interventions. We are encouraged that this intervention could have a substantial impact as an initial or adjunct treatment for patients with mild to moderate knee OA.

## Competing interests

Visual Health Information, Inc (VHI) owns the website used in the current manuscript, of which Beaulieu is the president/CEO and Cooper is the lead software engineer. VHI funded the article-processing charge associated with this manuscript. Severson and Heiderscheit have received consulting fees from VHI for work unrelated to the manuscript under review. A patent related to the content of the manuscript is currently under review.

## Authors’ contributions

AB participated in the study design and coordination and drafted the manuscript. JB conceived of the study, helped develop the website, participated in the study design and coordination, and helped draft the manuscript. HS participated in the study design and coordination, helped with statistical analysis, and helped draft the manuscript. CW participated in study coordination and helped draft the manuscript. JG performed the statistical analysis and helped draft the manuscript. BH participated in the study design and coordination and drafted the manuscript. All authors read and approved the final manuscript.

## Pre-publication history

The pre-publication history for this paper can be accessed here:

http://www.biomedcentral.com/1471-2474/15/158/prepub
